# Efficacy of intergrating vestibular rehabilitation and cognitive behaviour therapy in persons with persistent dizziness in primary care- a study protocol for a randomised controlled trial

**DOI:** 10.1186/s13063-019-3660-5

**Published:** 2019-10-07

**Authors:** Lene Kristiansen, L. H. Magnussen, K. T. Wilhelmsen, S. Mæland, S. H. G. Nordahl, R. Clendaniel, A. Hovland, B. Juul-Kristensen

**Affiliations:** 1 Department of Health and Functioning, Faculty of Health and Social Sciences, Western Norway University of Applied Sciences, Bergen, Norway; 2 Norwegian National Advisory Unit on Vestibular Disorders, Department of Otorhinolaryngology and Head Neck Surgery, Haukeland University Hospital, Bergen, Norway; 3 Department of Clinical Medicine, University of Bergen, Bergen, Norway; 4 Doctor of Physical Therapy Division, Department of Orthopedics, Duke University School of Medicine, Durham, NC USA; 5 Department of Clinical Psychology, University of Bergen, Bergen, Norway; 6 Solli District Psychiatric Centre (DPS), Nesttun, Norway; 7 Department of Sports Science and Clinical Biomechanics, University of Southern Denmark, Odense, Denmark

**Keywords:** Protocol, RCT, Persistent dizziness, Vestibular rehabilitation, Cognitive behaviour therapy, Dizziness Handicap Inventory, Gait velocity, Rehabilitation

## Abstract

**Background:**

Dizziness is a common complaint, and the symptom often persists, together with additional complaints. A treatment combining Vestibular Rehabilitation (VR) and Cognitive Behaviour Therapy (CBT) is suggested. However, further research is necessary to evaluate the efficacy of such an intervention. The objective of this paper is to present the design of a randomised controlled trial aiming at evaluating the efficacy of an integrated treatment of VR and CBT on dizziness, physical function, psychological complaints and quality of life in persons with persistent dizziness.

**Methods/design:**

The randomised controlled trial is an assessor-blinded, block-randomised, parallel-group design, with a 6- and 12-month follow-up. The study includes 125 participants from Bergen (Norway) and surrounding areas. Included participants present with persistent dizziness lasting for at least 3 months, triggered or exacerbated by movement. All participants receive a one-session treatment (Brief Intervention Vestibular Rehabilitation; BI-VR) with VR before being randomised into a control group or an intervention group. The intervention group will further be offered an eight-session treatment integrating VR and CBT. The primary outcomes in the study are the Dizziness Handicap Inventory and preferred gait velocity.

**Discussion:**

Previous studies combining these treatments have been of varying methodological quality, with small samples, and long-term effects have not been maintained. In addition, only the CBT has been administered in supervised sessions, with VR offered as home exercises. The current study focusses on the integrated treatment, a sufficiently powered sample size, and a standardised treatment programme evaluated by validated outcomes using a standardised assessment protocol.

**Trial registration:**

www.clinicaltrials.gov, ID: NCT02655575. Registered on 14 January 2016.

**Electronic supplementary material:**

The online version of this article (10.1186/s13063-019-3660-5) contains supplementary material, which is available to authorized users.

## Background

Vertigo and/or dizziness are amongst the most frequent symptoms reported in outpatient practices [1], with a lifetime prevalence reported in approximately 30% [2]. Dizziness can present for a variety of reasons, many initiated through vestibular disease; however, it is not always possible to identify a specific cause or diagnosis [3].

Psychological factors, such as anxiety, seem to be closely related to the prevalence of dizziness [4, 5], and it is likely that biological and psychological factors interact, maintaining the vestibular symptoms as well as the anxiety [4, 6]. In chronic dizziness it is common to avoid movements, activities and social settings that may provoke symptoms and discomfort [7]. This fear of provoking dizziness and discomfort may also lead to an ‘en bloc’ movement pattern. Some studies on persons with persistent dizziness have also reported musculoskeletal symptoms [8] like, for instance, postural malalignments [9] and musculoskeletal pain [10], particularly in the neck-shoulder area [10–12]. Long-term consequences of such avoidance strategies may hamper compensation strategies and functional improvements, eventually leading to occupational disability [13].

Vestibular Rehabilitation (VR) is an exercise-based treatment approach for dizziness, primarily directed towards reducing vestibular symptoms (not musculoskeletal aberrations), with moderate to strong evidence of VR for conditions of unilateral vestibular hypofunction [14]. A recent review has indicated that VR may also be used in other conditions such as vestibular disorders of central origin [15]. Since musculoskeletal symptoms are not specifically targeted in VR interventions, one longitudinal study (no control group) incorporated body-awareness therapy [16] into VR, with positive effects on musculoskeletal aberrations, such as improved bodily flexibility and balance during ambulation, as well as improved perception of dizziness [17, 18].

As mentioned, VR treatment is developed as an exercise-based treatment; however, it also contains some cognitive elements, such as graded exposure (habituation), that also facilitates cognitive restructuring (e.g. reduce avoidance behaviour) [7, 19]. A recent randomised controlled trial (RCT), providing just three sessions of Cognitive Behaviour Therapy (CBT) for panic anxiety, found reduced dizziness, handicap and use of safety behaviours in persons with chronic subjective dizziness [20]. Furthermore, the positive changes were maintained at 6-month follow-up [21], but the outcomes only focussed on psychological complaints, and no objective outcomes were assessed.

As both VR and CBT have shown positive effects on persons with dizziness, the combination of VR and CBT seems to be an appropriate treatment approach for persistent dizziness, and a few studies have investigated this combination [22–25]. The effects of the combined VR and CBT treatments are reported to be reduced dizziness-related handicap [23–25], improved walking [23], and reduced anxiety and depression [25]. A systematic review on psychotherapy in dizziness found a small and clinically relevant effect on dizziness, but no effect on anxiety and depression [26]. However, the included studies had small sample sizes (19 to 31 participants) [23–25], no random allocation [25], no standardised CBT treatment manual [25], and improvements found in the short-term were not maintained as long-term effects [22]. Further, as none of the combined treatments included a focus on musculoskeletal complaints, there is a need to further develop the treatment combining VR and CBT, also targeting the musculoskeletal aspects, and afterward the effects of the programme must be tested in a RCT. A recent feasibility study integrating VR and CBT with an additional focus on musculoskeletal effects showed that such a treatment approach was feasible and safe [27]. Therefore, this treatment is now ready to be evaluated in a RCT.

### Study objectives

The aim of the RCT is to evaluate the short- (6 months) and long-term (12 months) efficacy of an integrated treatment of VR and CBT in persons with persistent dizziness. It is hypothesised that persons receiving the additional Vestibular Rehabilitation and Cognitive Behaviour Therapy (VR-CBT) programme will show superior reduction in self-reported dizziness-related handicap in addition to increased preferred gait velocity compared with persons receiving Brief Intervention Vestibular Rehabilitation (BI-VR) alone.

## Methods/design

### Study design

The study is a prospective, assessor-blinded, block-RCT, with a parallel-group design, with a 6- and 12-month follow-up. The protocol conforms with the recommendations from the EQUATOR network [28], using the Standard Protocol Items: Recommendations for Interventional Trails (SPIRIT) Checklist and the Consolidated Standard of Reporting Trials (CONSORT) guidelines when reporting the results [29, 30]. The SPIRIT Checklist is available in Additional file 1.

### Settings and location

Participants will be recruited through general practitioners, physiotherapists, ear, nose and throat specialists, and information through newspapers and social media. Participants will be recruited from the region in and around Bergen, Norway. Blinded baseline and follow-up testing and the one-session VR intervention (BI-VR) will be conducted at the Western Norway University of Applied Sciences (HVL). Group treatment (VR-CBT) will be offered at HVL, as well as at selected physiotherapy clinics in Bergen. The group treatment will be led by physiotherapists trained in the treatment protocol (please see below).

### Participants

Eligible participants must meet all the following inclusion criteria: be of working age (18–70 years) with acute onset of dizziness and with symptoms lasting at least 3 months, and the dizziness has to be triggered/worsened by head movements,

Participants will be excluded if they meet one or more of the following exclusion criteria: self-reported non-vestibular reason for dizziness (e.g. neurological conditions) or fluctuating vestibular diseases (e.g. Ménière’s disease); scheduled for treatment of/have had treatment for benign paroxysmal positional vertigo (BPPV) within 1 month; fast head movements are contraindicated (e.g. whiplash-associated injuries, osteoporosis of the neck); presentation of severe/terminal pathology (cancer, psychiatric diagnosis); participation in group therapy for dizziness within the past year; inadequate Norwegian language proficiency (verbal and written); or unable to attend test and treatment locations.

### Procedure

Eligible participants will initially be screened by a telephone interview followed by further screening at HVL. Participants fulfilling the inclusion and exclusion criteria who are willing to participate will be asked to sign an informed consent (Additional file 2, in Norwegian). The first meeting comprises screening by the assessors, signing of informed consent, and baseline testing. This is scheduled to last 2 h. During baseline testing the included participants will complete physical tests and questionnaires. Following baseline testing the participants attend a 1-h treatment session (BI-VR), and afterwards randomisation to either the intervention group or the control group. Follow-up testing is scheduled 6 and 12 months after inclusion (Table 1) comprising online or paper versions of the questionnaires (completed separately from testing) and objective outcomes. Both follow-up tests are scheduled to last up to 1 h.

**Table 1:** Standard Protocol Items: Recommendations for Interventional Trials (SPIRIT) Figure of study protocol

	Study period				
	Enrolment			Post allocation	
Timepoint		Month 0-2	Month 1-6	6 months	12 months
Enrolment					
Eligibility screen	X				
Informed consent	X				
Allocation		X			
Interventions					
BI-VR		X			
VR-CBT			X		
Assessments					
Dizziness Handicap Inventory	X			X	X
Preferred gait velocity	X			X	X
Vertigo Symptom scale- Short form	X			X	X
Body Sensation Questionnaire	X			X	X
Adapted Panic attack Scale	X			X	X
Mobility Index, Alone	X			X	X
Panic attack scale	X			X	X
Patient Specific function questionnaire	X			X	X
Subjective Health complaints	X			X	X
Patient global impression of change				X	X
Chalders fatigue questionnaire	X			X	X
EQ5D-5L	X			X	X
Body sway in standing	X			X	X
Head movement induced dizziness	X			X	X
Fast gait velocity	X			X	X
Clinical dynamic visual acuity	X			X	X
Elements from GPE	X			X	X
Dual task walking	X			X	X
Grip strength	X			X	x

### Interventions

#### Brief Intervention Vestibular Rehabilitation

All the participants receive BI-VR, a single-session treatment based on elements from traditional VR [31, 32], but adapted to a single session in line with a brief intervention model developed for patients with low back pain [33]. The purpose of the treatment is to give the participant the understanding that movement is the key factor in improving symptoms and that dizziness is rarely related to serious illness. BI-VR comprises examination, information regarding the vestibular system, what causes dizziness, advice related to specific findings, and supervision in selected, standardised VR exercises. All participants are encouraged to stay active, and provoke dizziness in line with established recommendations [14, 34].

### Control group

Participants allocated to BI-VR only will be encouraged to do the prescribed exercises on their own. The BI-VR physiotherapist will call twice during a 4-month period, to encourage compliance with the home exercises and answer questions that may arise.

### Intervention group

#### Vestibular Rehabilitation and Cognitive Behavioural Therapy

Participants in the intervention group will be invited to attend an additional structured group-treatment programme integrating Vestibular Rehabilitation and Cognitive Behaviour Therapy (VR-CBT). The VR-CBT manual was developed through collaboration between researchers, physiotherapists and clinical psychologists. The treatment offers eight weekly 2-h sessions with five to eight participants in each group, with the aim of addressing both the physical and psychological challenges of persistent dizziness. The CBT approach is based on previous findings indicating that treatment for panic disorders can also be efficacious for persons with chronic dizziness [20]. CBT focusses on the vicious cycle between somatic anxiety symptoms elicited by the ‘fight or flight’ response, the catastrophic misinterpretations of these and other bodily symptoms, and the resulting safety-seeking and avoidance behaviour [35]. The VR comprises habituation, gaze stability and balance exercises, with body awareness promoted throughout, in addition to guided relaxation [34, 36]. The exercises may be individually adapted by, for instance, adjustments in base of support, speed of movement, and environmental conditions. All sessions will have elements of both VR and CBT; however, the first three sessions mostly emphasise CBT, while the subsequent five sessions mostly emphasise VR. This set-up allows the participants to practise exercises in a safe environment, and provides opportunities to reflect on dizziness, safety and avoidance behaviours that may occur. Participants are further asked to carry out and register home exercises following the treatment sessions, and daily VR exercises are introduced from session 3 onwards. A brief description of the VR-CBT manual is presented in Table 2.

**Table 2:** Brief description of the Vestibular Rehabilitation and Cognitive Behaviour Therapy (VR-CBT) group-treatment protocol

Session number	Focus	Example of tasks/exercises
1	Dizziness and additional/secondary complaints	Discussion on dizziness and additional complaints. Introducing the vicious circle that can arise between somatic symptoms and the catastrophic misinterpretation of these. Exercises: bdy awareness in sitting and standing. Habituation (nodding and head turns)
2	The ‘vicious circle’	How somatic symptoms related to both dizziness and anxiety can be appraised appropriately by mapping the relevant symptoms, thoughts and potential avoidance behaviour for each participant. Introducing the ‘fight or flight’ response, and how this may be relevant for chronic dizziness. Exercises: body awareness in standing and walking, habituation through games with planned and unplanned head turns. Relaxation
3	The fight or flight response	Discussion regarding experiences related to symptoms similar to the fight or flight response. How can these symptoms be appraised in relation to persistent dizziness? Exercises: habituation and body awareness (standing balance, walking with directional changes). Reflection during and after exercises. What happened? What was your response? (every session from now). Relaxation
4	The fight or flight response and management	Discussion: how did you respond to the fight or flight response in everyday life following the last session? Individual goal setting. Exercises: habituation, visual acuity, walking and ball games with change of place, turning and rotation. Relaxation
5	Relaxation	Discussion/reflection: exercises, dosage and ‘relaxation’. It is normal to be dizzy and tired after exercises Exercises: progression of visual acuity, habituation and balance using ball during exercise. Working alone and in pairs. Relaxation
6	Movement-induced dizziness	Any changes in relation to the dizziness circle described in the first session? Group and individual reflection. Exercises: habituation games: in larger groups and pairs. Walking with head rotations, velocity changes and externally induced stop/start. Relaxation
7	What next? Preparation for the future	Discussion before, reflection during, and group reflection after exercises: ‘How do I cope/deal/manage the dizziness? What thoughts are formed when I get dizzy?’ Exercises: combination of balance and habituation – Activities and games in groups and in pairs. (e.g. obstacle course, standing back to back, passing ball at different heights.). Relaxation
8	Reflection and conclusion	Discussion: ‘What have I learnt? What will I take with me? What do I do when/if dizziness returns?’ Exercises: balance and body awareness in standing and walking, changing directions, different velocities, stop/start. Ball activities alone, in pairs and in a larger group. Relaxation

### Physiotherapists

One physiotherapist experienced in VR, and trained in the BI-VR protocol, will run all BI-VR sessions. Six physiotherapists delivering the VR-CBT treatment will attend a competency course to before leading the treatment. The competency course contains the principles of VR-CBT, the elements of the treatment manual, and training of practical skills related to the manual, as described in the feasibility study [27]. After each of the first two treatment sessions a clinical psychologist and the principal investigator will be available for support and guidance, without unblinding participant allocation.

### Data collection and follow-up

Three assessors (principal investigator, project lead and one research assistant) are involved in collecting informed consent, and blinded data collection at baseline and follow-up, adhering to the standardised test protocol. In addition, the assessors will practise together before and during data collection, in order to unify performance and interpretation of the outcome measures. The principal investigator will perform the majority of the data collection.

Personal information related to the participants will be stored on a secure server only accessible to the researchers involved in the research projects. Outcomes collected on paper will be registered into a secure file and placed on the same server, and paper copies will be kept in a locked cupboard only accessible by the principle investigator. After completion of the project all paper copies will be destroyed, and the dataset will be anonymised.

### Outcome measures

Table 3 describes the outcomes that will be collected at the various stages in the study.

**Table 3:** Description and test metrics of outcome measures

Name	Scoring/description	Test metrics
Primary outcome measures		
Dizziness Handicap Inventory (DHI)	25 items, each item has 3 alternative scores 0 (no), 2 (sometimes) and 4 (yes) giving a score range of 0–100 DHI points [37]. Higher scores indicate greater perceived disability; 0–30; mild, 31–60; moderate, 61–100; severe [38]	Cut-off 29 points, MIC 11 DHI points, ICC 1,1 0.90 [39].
Preferred gait velocity (m/s)	Participants walked at normal pace, down an 8-m pathway, timed in the middle 6 m. It was timed using a stopwatch from when the first foot passed the start point to when the last foot passed the stop point. Mean velocity over two trials were calculated	Substantial meaningful change 0.1 m/s [40], ICC (3.1): 0.88 (CI 0.81–0.98) [41]
Secondary outcomes/patient-reported outcomes		
The shortened version of the Vertigo Symptom Scale (VSS)	15 items, each scoring from 0 (never) to 4 (very often) giving a score range of 0–60. Higher scores indicate greater symptom severity [42]. Severe dizziness ≥ 12 [42]	Norwegian version cut-off, 6.5 points [43]. Clinically significant change in original version ≥ 3 points [44]. ICC Norwegian version, 0.89 [43]
Agoraphobic Cognitions Questionnaire (ACQ)	14 items, each rated on a scale ranging from 1 (thought never occurs when I am nervous) to 5 (thought always occurs when I am nervous) [45]. Measures fear of fear. The mean score is reported, and higher scores imply greater levels of fear	Cronbach’s alpha for outpatients with agoraphobia, 0.80 [45]
Body Sensation Questionnaire (BSC)	18 items, each with a score range from 1 (not at all frightened by the sensations) to 5 (extremely frightened by this sensation). The mean score reported, and higher scores implies greater fear of somatic sensations [45]	Cronbach’s alpha for outpatients with agoraphobia, 0.87 [45]
Mobility Inventory of Agoraphobia-Alone (MIA)	27 items, each rated from 1 (never avoids) to 5 (always avoids). The mean score is reported and, and higher scores indicate greater avoidance behaviour	Cronbach’s alpha in agoraphobia, 0.96 [46]
Adapted Panic Attack Scale		
• Attack frequency	Measures frequency of distress related to sudden onsets of episodes with 4 or more strong sensations of dizziness and dizziness related symptoms on a 5-point scale ranging from 0 (no attacks) to 4 (one or more attacks per day). Adapted from the Panic Attack Scale [47]	
• Attack severity	Severity rating of the degree of distress related to the episodes described above. Numeric rating scale with a score range 0–8. Higher scores indicates increased symptom-related distress/disability. Adapted from the Panic Attack Scale [47]	
Hospital Anxiety and Depression Scale (HADS)	14 items, each rated from 0 (not present) to 3 (considerable), giving a score range of 0–42 points [48]. Higher scores indicates greater psychological distress	Cut-off 12 points, Cronbach’s alpha, 0.88. [49]
EQ-5D-5 L	Generic instrument describing and valuing health [50].	
• EQ-5D-5 L	Five dimensions, each rated from 1 to 5. Higher scores indicate increased health problems [51]	
• EQ-5D-5 L Vas	Score range 0–100%. Higher scores indicate better perceived health-related quality of life	MCID in stroke,:8.61–10.82 [52]
Subjective Health Complaints (SHC)	29 items, each item is scored from 0 (no complaints) to 3 (serious complaints). Higher scores indicate greater severity of complaint. Split into 5 subcategories: Musculoskeletal 8 items (score 0–24), Pseudoneurology 7 items (score 0–21), Gastrointestinal 7 items (score 0–21), Flu 2 items (score 0–6) and Allergy 5 items (score 0–15) [53]	Cronbach’s alpha musculoskeletal pain, 0.74; Pseudoneurology, 0.73; Gastrointestinal, 0.62; Allergy, 0.58; and Flu, 0.67 [53]
Chalder’s Fatigue Questionnaire (CFQ)	13 items. The first 11 items are scored from 0 (better than usual) to 3 (much worse than usual), giving a score range of 0–33. The last 2 items rate duration and constancy of fatigue [54]. Higher scores indicating more fatigued	Cronbach’s alpha in Norwegian population, 0.86 [55]
Patient Specific Functional Scale (PSFS)	Registers up to 3 activities that participants find difficult. In addition, the level of difficulty is rated on an 11-point scale [56], where 0 maximum difficulty and 10 is no difficulty	Reliability established in various musculoskeletal problems (ICC 0.76–0.97) [57] MCID in various musculoskeletal problems, 0.99–2.5 [57]
Patient Global Impression of Change (PCIG)	1item, rated from 1 (very much improved) to 7 (very much worse), with a score of 4 indicating no change [58]	
Secondary outcomes/physical tests		
Dual-task walking	Similar walking protocol as for preferred gait velocity, with an added task of counting backwards by 3 out loud, while walking. Each trial was timed and the numbers of miscounts were documented. Mean velocity, and mistakes over 2 trials calculated	
Fast gait velocity (m/s)	Similar protocol to preferred gait velocity; however, participants were asked to walk as fast as possible	
Clinical dynamic visual acuity (CDVA)	Evaluates gaze stability by assessing visual acuity using examiner-mediated head oscillations at 2 Hz relative to head being stationary	Cut-off ≥ 3 lines indicates potential vestibular hypofunction [59]. Reliability in bilateral peripheral hypofunction ICC (2.2): 0.94 [60]
Head-movement-induced dizziness	Perceived dizziness reported using the Numeric Rating Scale (NRS) on 2 conditions: 1 while sitting stationary, and 1 after 1 min of active head oscillations at 1 Hz (following a metronome). Score range 0 (no dizziness) to 10 (as bad as it can be), with higher scores indicating higher perceived intensity of head-movement-induced dizziness. Difference between the two conditions will also be calculated	VAS head-movement-induced dizziness [41], reliability 0.48 for all subjects, reliability 0.82 for male subjects
Grip strength	Maximal grip strength in both hands assessed using a hand-held dynamometer. Measured in kg. Averaged between 2 trials calculated for each hand	Genuine change in healthy adults, 6 kg [61]
Body sway while standing	Assessed using the modified test for interaction and balance (mCTSIB) with arms crossed over the chest, using the HURlabs balance trainer BTG4; 4 conditions tested: standing with eyes open and closed, on a firm surface or on a foam cushion. Each trial is timed for 30 s	ICC in healthy subjects, 0.91–0.97 [62]
Elements from the Global Physiotherapy Examination (GPE)	4 elements from the main domain Movement of the GPE examination were selected [17, 36]. The items include lumbo-sacral flexion, head-nod flexion, shoulder retraction and elbow drop. Score range − 2.3 to 2.3, scored in relation to a predefined standard (0) [36]	ICC 2.1 lumbo-sacral flexion, 0.82; ICC 2.1 head-nod flexion, 0.84; ICC 2.1 shoulder retraction, 0.75; ICC 2.1 elbow drop, 0.89 (personal communication: A. Kvåle)

Abbreviations: *ICC* Intercal correlation coefficient, *MCID* minimal clinical important difference, *mCTSIB* Modified test for sensory interaction and balance, *MIC* minimal important change

### Primary outcome measures

The primary outcome measures are the Dizziness Handicap Inventory (DHI) and preferred gait velocity. The DHI is a questionnaire developed in order to assess the impact of dizziness on quality of life (QoL) [37]. It is translated into Norwegian and has shown satisfactory test-retest reliability [39]. Preferred gait velocity is assessed using the 6-m timed gait test, with one additional metre at each end allowing acceleration and deceleration [41]. The test has been found to be reliable in healthy adults [63], as well as in persons with vestibular disorders [41].

### Secondary outcome measures

The secondary outcomes include dizziness severity, psychological complaints, fatigue, subjective health complaints, standing balance, walking, strength, flexibility and general QOL.

The patient-reported outcomes is used to evaluate dizziness severity using the Norwegian version [43] of the Vertigo Symptom Scale short form (VSS) [42]. In addition, the Hospital Anxiety and Depression Questionnaire (HADS) [48], the Body Sensations Questionnaire (BSQ) [45], the Agoraphobic Cognition Questionnaire (ACQ) [45], the Mobility Inventory of Agoraphobia-Alone (MIA) [46], and an adapted version of the Panic Attack Scale (PAS) [47] describes levels of anxiety, depression, panic-related symptoms and avoidance behaviour. Further, fatigue is assessed using the Chalder’s Fatigue Questionnaire (CFQ) [54], while the Subjective Health Complaints (SHC) inventory reports incidents and extent of subjective somatic and psychological complaints [64]. The Patient Specific Functional Scale (PSFS) assesses perceived functional change [56], while information regarding QOL is gathered using the EuroQol five-dimension, five-level health survey (EQ-5D-5 L) [65].

Secondary objective measures include standing balance (sway measured during the four conditions in the modified clinical test for sensory interaction and balance (mCTSIB)) [66] using balance trainer BTG4 (HUR health, Kokkola, Norway), walking (fast velocity and with dual task [67]), visual acuity (Clinical Dynamic Visual Acuity Test (CDVA) [59]) and grip strength [68]. Musculoskeletal aberrations are registered using four elements from the Global Physiotherapy Examination (GPE) [17] and head-movement-induced dizziness [41] measured using the numeric rating scale (HmDizz). The Patient Global Impression of Change questionnaire (PGIC) [58] is used to evaluate perceived improvement at follow-up testing at 6 and 12 months.

### Demographic data and other measurements

In addition to the outcomes, information regarding gender, age, work status, medication and activity level will be gathered. The VR-CBT physiotherapists will register attendance to all sessions and reasons for absence and collect the home-exercise registrations.

Satisfactory compliance to VR-CBT will be defined as minimum 75% attendance to VR-CBT sessions (six out of eight sessions), and minimum 80% completion of the exercise diary for home exercises, where 100% completion is defined by reporting exercises and following a walking programme five times per week*.* Satisfactory compliance in the control group will be defined as completion of at least one telephone call with the BI-VR physiotherapist.

### Sample size and power considerations

The study is designed as a RCT comparing two groups (BI-VR, and BI-VR with VR-CBT). To obtain a clinically important group difference in DHI of 11 points [39] with a significance level of 0.05 and a power of 80%, 47 participants will be required per group. To obtain a clinically important change in preferred gait velocity of 0.1 m/s [69] with a significance level of 0.05 and a power of 80%, 36 participants will be required in each group. In order to ensure power of both primary outcomes at least 47 participants are selected as the basis for the sample size needed in the study. The final sample size is set at 125 participants, allowing for an approximately 35% drop-out, based on drop-outs in the feasibility study [27] and in previous studies [18, 20, 70, 71].

### Randomisation and concealment of allocation

The participants are block-randomised in groups of 16, and randomly assigned to BI-VR followed by VR-CBT (intervention group) or BI-VR alone (control group). Group allocation is performed using a random number generator and is presented on a folded paper, in a concealed envelope. The principle investigator is blinded from group allocations. The envelopes are stored in a locked cupboard only accessible to the BI-VR physiotherapist handing out the allocation envelopes. After group allocation the VR-CBT participants will be contacted by the project lead regarding the first VR-CBT appointment.

### Blinding

The principal investigator and assessors are blinded from group allocation and not involved in the treatment of the participants. Blinding of group allocation for VR-CBT physiotherapists and participants is not possible. However, both groups are informed that the optimal treatment is not known, and the study hypothesis is not presented. In order to ensure blinding of assessors the participants are encouraged not to reveal their allocation during testing.

### Statistical analysis plan

The efficacy analysis is assessment of the between-group differences in changes in DHI score and preferred gait velocity at 6- and 12-month follow-up. The analysis will use the intention-to-treat (ITT) principle, analysing all randomised participants independent of compliance and withdrawals. In the event of missing data two methods will be used. For missing single questions, the mean baseline value for the respected group will be assigned. If complete questionnaires or objective measures are missing a non-responder imputation will be used, including baseline data carried forward. Sensitivity analyses will be performed to study whether those who drop out differ from those who complete the required programme.

An analysis of covariance (ANCOVA) will be used to analyse mean changes in continuous variables, and logistic regression for categorical variables. The model will include the respective dependent variable, in addition to fixed effects of group allocation, baseline value, age, gender and height.

The results will be expressed as a difference between the group means and 95% confidence intervals with associated *p* values. The main analyses will be conducted by a statistician not involved in the testing and blinded to group allocation. All data analysis will be performed according to a pre-established statistical analysis plan and interpreted according to a consensus document signed by all authors. All analyses will be performed using Statistical Package for Social Sciences (SPSS) (version 25, IBM, Armonk, NY, USA).

#### Interim analyses

Drop-out rates will be assessed in interim analyses to determine the potential need for adjustment in sample size in line with the power calculations.

### Ethical considerations

Although participants may experience increased symptoms in the short term, it is not anticipated that participation will cause any serious adverse events or harms. A recent feasibility study [27] has confirmed that the intervention BI-VR and VR-CBT is feasible and safe for persons with persistent dizziness. If participants experience harm from the treatment delivered within the study, this will be registered, and the person will be directed for further assessment and follow-up care in accordance with the provisions that are customary within the national, public health care system. The study will follow the criteria and principles of the Declaration of Helsinki. It has been approved by the Regional Committee for Medical and Health Research Ethics (2014–00921) and is registered at www.clinicaltrials.gov (NCT02655575).

## Discussion

In recent years there has been more focus on including cognitive approaches into the treatment of persistent dizziness [20, 72]. However, as previously mentioned there are only a few studies conducted on the effect of combining VR and CBT in persons with persistent dizziness [23–25]. All of the studies have scientific limitations as mentioned, categorising them as moderate quality. The promising result of the recent studies, and the lack of high-quality studies have shown the need for further studies on the efficacy of combining CBT and VR for participants with persistent dizziness.

The novelty of the current study is the integration of VR and CBT, with an additional focus on musculoskeletal complaints, as treatment for persons with persistent dizziness. The previous studies conducted CBT as supervised sessions (individually [25] or in small groups [23, 24]) while VR exercises where administered as home exercises [24, 25]. The integration of the supervised VR-CBT sessions in the current RCT allows the participants to practise exercises in a safe environment supervised by physiotherapists, and the exercises may be adapted to meet the level of complaints and capacity in each participant.

One possible limitation in the study, as with all exercise trials, is the inability to blind physiotherapists and participants to treatment allocation. Thus, only the testers can be blinded to group allocation. Another possible limitation includes the specification of compliance criteria, which to our knowledge has not been done in this research area before, and there is no consensus regarding how much is sufficient. Also, a block-randomisation of 16 may be seen as a limitation as it may take time before the group number is reached. In addition, confounding factors like, for instance, age, gender, duration of complaints and psychological factors, may have an impact on primary and secondary outcomes. However, the randomised design is expected to distribute these variables equally between the allocation groups.

The strength of the current study is the inclusion of relevant reliable main outcomes with a sufficiently powered sample size. In addition, the study utilises standardised testing procedures, evaluating both short- and long-term efficacy. Another strength is the initial feasibility study conducted, showing that the test procedures and interventions were feasible and sage for the present population [27]. To our knowledge, the current RCT study is the largest study to date, combining VR and CBT to treat persons with persistent dizziness.

The aim of the current study is to evaluate the efficacy of BI-VR followed by VR-CBT, compared with BI-VR alone. If the treatment group improves more than the control group, the description of a standardised programme may help practitioners to treat persons with persistent dizziness. If there is no difference between the groups, it may indicate that persons with persistent dizziness may manage their complaints with minimal support from physiotherapists.

## Trial status and publication plan

Recruitment of participants to the main study, applying version 2 (dated 30 January 2016) of the study protocol, started on 1 February 2016 and is expected to end by 1 May 2019. At the time of submission of this protocol (April 2019) the trial is ongoing and still recruiting. Currently, 106 participants have been included. When recruitment is finished the data will be analysed, interpreted and published regardless of positive, negative or inconclusive results.

## Additional files

Additional file 1: Standard Protocol Items: Recommendations for Interventional Trials (SPIRIT) 2013 Checklist: recommended items to address in a clinical trial protocol and related documents*. (DOC 137 kb)

Additional file 2: Consent form for participation, in Norwegian (DOC 48 kb)

## Data Availability

Not applicable.

## Abbreviations

ACQ: Agoraphobic Cognitions Questionnaire
ANCOVA: Analysis of covariance
BI-VR: Brief Intervention Vestibular Rehabilitation
BPPV: Benign paroxysmal positional vertigo
BSQ: Body Sensation Questionnaire
CBT: Cognitive Behaviour Therapy
CDVA: Clinical Dynamic Visual Acuity
CFQ: Chalder’s Fatigue Questionnaire
DHI: Dizziness Handicap Inventory
DTW: Dual-task walking
GP: General practitioner
GPE: Global Physiotherapy Examination
HADS: Hospital Anxiety and Depression Scale
mCTSIB: Modified test for sensory interaction and balance
MIA: Mobility Index of Agoraphobia-Alone
PAS: Panic Attack Scale
QOL: Quality of life
SHC: Subjective Health Complaints
VR: Vestibular Rehabilitation
VR-CBT: Vestibular Rehabilitation and Cognitive Behaviour Therapy
VSS: Vertigo Symptom Scale short form

## References

[1] Grill E, Strupp M, Müller M, Jahn K (2014). Health services utilization of patients with vertigo in primary care: a retrospective cohort study. J Neurol.

[2] Neuhauser HK (2007). Epidemiology of vertigo. Curr Opin Neurol.

[3] Bösner S, Schwarm S, Grevenrath P, Schmidt L, Hörner K, Beidatsch D (2018). Prevalence, aetiologies and prognosis of the symptom dizziness in primary care–a systematic review. BMC Fam Pract.

[4] Yardley L, Redfern MS (2001). Psychological factors influencing recovery from balance disorders. J Anxiety Disord.

[5] Staab JP (2011). Behavioral aspects of vestibular rehabilitation. NeuroRehabilitation..

[6] Guidetti G (2013). The role of cognitive processes in vestibular disorders. Hear Balance Commun.

[7] Yardley L, Beech S, Weinman J (2001). Influence of beliefs about the consequences of dizziness on handicap in people with dizziness, and the effect of therapy on beliefs. JPsychosomRes..

[8] Pavlou M, Newham D, Bronstein AM (2013). The principles of balance treatment and rehabilitation. The Oxford textbook of vertigo and imbalance.

[9] Coelho Júnior AN, Gazzola JM, Gabilan YP, Mazzetti KR, Perracini MR, Gananca FF (2010). Head and shoulder alignment among patients with unilateral vestibular hypofunction. RevBrasFisioter..

[10] Iglebekk W, Tjell C, Borenstein P (2013). Pain and other symptoms in patients with chronic benign paroxysmal positional vertigo (BPPV). Scand J Pain.

[11] Tamber AL, Bruusgaard D (2009). Self-reported faintness or dizziness—comorbidity and use of medicines. An epidemiological study. Scand J Public Health.

[12] Wilhelmsen K, Ljunggren AE, Goplen F, Eide GE, Nordahl SH (2009). Long-term symptoms in dizzy patients examined in a university clinic. BMCEar Nose Throat Disord.

[13] Skoien AK, Wilhemsen K, Gjesdal S (2008). Occupational disability caused by dizziness and vertigo: a register-based prospective study. BrJGenPract..

[14] McDonnell MN, Hillier SL (2015). Vestibular rehabilitation for unilateral peripheral vestibular dysfunction. Cochrane Database Syst Rev.

[15] Dunlap PM, Holmberg JM, Whitney SL (2019). Vestibular rehabilitation: advances in peripheral and central vestibular disorders. Curr Opin Neurol.

[16] Kvåle A, Ljunggren AE, Schmidt RF, Willis WD (2007). Body awareness therapies. Encyclopedia of pain. Springer Reference.

[17] Kvale A, Wilhelmsen K, Fiske HA (2008). Physical findings in patients with dizziness undergoing a group exercise programme. Physiother Res Int.

[18] Wilhelmsen K, Nordahl SH, Moe-Nilssen R (2010). Attenuation of trunk acceleration during walking in patients with unilateral vestibular deficiencies. JVestibRes..

[19] Beidel DC, Horak FB (2001). Behavior therapy for vestibular rehabilitation. J Anxiety Disord.

[20] Edelman S, Mahoney AE, Cremer PD (2012). Cognitive behavior therapy for chronic subjective dizziness: a randomized, controlled trial. Am J Otolaryngol.

[21] Mahoney AEJ, Edelman S, Cremer PD (2013). Cognitive behavior therapy for chronic subjective dizziness: longer-term gains and predictors of disability. Am J Otolaryngol.

[22] Holmberg J, Karlberg M, Harlacher U, Magnusson M (2007). One-year follow-up of cognitive behavioral therapy for phobic postural vertigo. JNeurol..

[23] Johansson M, Akerlund D, Larsen HC, Andersson G (2001). Randomized controlled trial of vestibular rehabilitation combined with cognitive-behavioral therapy for dizziness in older people. Otolaryngol Head Neck Surg.

[24] Andersson G, Asmundson GJ, Denev J, Nilsson J, Larsen HC (2006). A controlled trial of cognitive-behavior therapy combined with vestibular rehabilitation in the treatment of dizziness. Behav Res Ther.

[25] Holmberg J, Karlberg M, Harlacher U, Rivano-Fischer M, Magnusson M (2006). Treatment of phobic postural vertigo. A controlled study of cognitive-behavioral therapy and self-controlled desensitization. J Neurol.

[26] Schmid G, Henningsen P, Dieterich M, Sattel H, Lahmann C (2011). Psychotherapy in dizziness: a systematic review. J Neurol Neurosurg Psychiatry.

[27] Kristiansen L, Magnussen L, Juul-Kristensen B, Mæland S, Nordahl S, Hovland A (2019). Feasibility of integrating vestibular rehabilitation and cognitive behaviour therapy for people with persistent dizziness. Pilot Feasibility Stud.

[28] Altman DG, Simera I, Hoey J, Moher D, Schulz K (2008). EQUATOR: reporting guidelines for health research. Lancet.

[29] Schulz KF, Altman DG, Moher D (2010). CONSORT 2010 Statement: updated guidelines for reporting parallel group randomised trials. BMC Med.

[30] Chan AW, Tetzlaff JM, Altman DG, Laupacis A, Gotzsche PC, Krleza-Jeric K (2013). SPIRIT 2013 Statement: defining standard protocol items for clinical trials. Ann Intern Med.

[31] Cawthorne T (1945). The physiological basis for head exersices. J Chartered Soc Physiother.

[32] Cooksey FS (1946). Rehabilitation in vestibular injuries. Proc R Soc Med.

[33] Indahl A, Haldorsen EH, Holm S, Reikerås O, Ursin H (1998). Five-year follow-up study of a controlled clinical trial using light mobilization and an informative approach to low back pain. Spine..

[34] Hall CD, Herdman SJ, Whitney SL, Cass SP, Clendaniel RA, Fife TD (2016). Vestibular rehabilitation for peripheral vestibular hypofunction: an evidence-based clinical practice guideline: from the American Physical Therapy Association neurology section. J Neurol Phys Ther.

[35] Clark DM (1986). A cognitive approach to panic. Behav Res Ther.

[36] Wilhelmsen K, Kvale A (2014). Examination and treatment of patients with unilateral vestibular damage, with focus on the musculoskeletal system: a case series. Phys Ther.

[37] Jacobson GP, Newman CW (1990). The development of the Dizziness Handicap Inventory. ArchOtolaryngolHead Neck Surg.

[38] Whitney SL, Wrisley DM, Brown KE, Furman JM (2004). Is perception of handicap related to functional performance in persons with vestibular dysfunction. OtolNeurotol..

[39] Tamber AL, Wilhelmsen KT, Strand LI (2009). Measurement properties of the Dizziness Handicap Inventory by cross-sectional and longitudinal designs. Health Qual Life Outcomes.

[40] Perera S, Mody SH, Woodman RC, Studenski SA (2006). Meaningful change and responsiveness in common physical performance measures in older adults. J Am Geriatr Soc.

[41] Hall CD, Herdman SJ (2006). Reliability of clinical measures used to assess patients with peripheral vestibular disorders. JNeurolPhysTher..

[42] Yardley L, Masson E, Verschuur C, Haacke N, Luxon L (1992). Symptoms, anxiety and handicap in dizzy patients: development of the Vertigo Symptom Scale. JPsychosomRes..

[43] Wilhelmsen K, Strand LI, Nordahl SH, Eide GE, Ljunggren AE (2008). Psychometric properties of the Vertigo Symptom Scale–Short form. BMCEar Nose Throat Disord.

[44] Yardley L, Donovan-Hall M, Smith HE, Walsh BM, Mullee M, Bronstein AM (2004). Effectiveness of primary care-based vestibular rehabilitation for chronic dizziness. AnnInternMed..

[45] Chambless DL, Caputo GC, Bright P, Galalgher R. Assessment of fear in agoraphobics: The Body Sensations Questionnaire and the Agoraphobic Cognitions Questionnaire. J Consult Clin Psychol. 1984;52(6):1090–97. 10.1037//0022-006x.52.6.1090 6520279

[46] Chambless DL, Caputo GC, Jasin SE, Gracely EJ, Williams C (1985). The Mobility Inventory for Agoraphobia. Behav Res Ther.

[47] Clark DM, Salkovskis PM, Hackmann A, Middleton H, Anastasiades P, Gelder M (1994). A comparison of cognitive therapy, applied relaxation and imipramine in the treatment of panic disorder. Br J Psychiatry.

[48] Zigmond A, Snaith R (1983). The Hospital Anxiety and Depression Scale. Acta Psychiatr Scand.

[49] Piker EG, Kaylie DM, Garrison D, Tucci DL (2015). Hospital Anxiety and Depression Scale: factor structure, internal consistency and convergent validity in patients with dizziness. Audiol Neurotol.

[50] Brooks R, Group E (1996). EuroQol: the current state of play. Health policy.

[51] Van Reenen M, Janssen B. EQ-5D-5L User guide: basic information on how to use the EQ-5D-5L instrument. http://Euroqol.org: Euroqol; 2015. Accessed 4 Feb 2019.

[52] Chen P, Lin K-C, Liing R-J, Wu C-Y, Chen C-L, Chang K-C (2016). Validity, responsiveness, and minimal clinically important difference of EQ-5D-5L in stroke patients undergoing rehabilitation. Qual Life Res.

[53] Eriksen HR, Ihlebaek C, Ursin H (1999). A scoring system for subjective health complaints (SHC). Scand J Public Health..

[54] Chalder T, Berelowitz G, Pawlikowska T, Watts L, Wessely S, Wright D (1993). Development of a fatigue scale. J Psychosom Res.

[55] Loge JH, Ekeberg Ø, Kaasa S (1998). Fatigue in the general Norwegian population: normative data and associations. J Psychosom Res.

[56] Stratford P, Gill C, Westaway M, Binkley J (1995). Assessing disability and change on individual patients: a report of a patient specific measure. Physiother Can.

[57] Horn KK, Jennings S, Richardson G, van Vliet D, Hefford C, Abbott JH (2012). The Patient-specific Functional Scale: psychometrics, clinimetrics, and application as a clinical outcome measure. J Orthop Sports Phys Ther.

[58] Hurst H, Bolton J (2004). Assessing the clinical significance of change scores recorded on subjective outcome measures. J Manip Physiol Ther.

[59] Tusa RJ, Herdman S, Clendaniel RA (2014). History and clinical examination. Vestibular rehabilitation. 4th ed.

[60] Rine RM, Braswell J (2003). A clinical test of dynamic visual acuity for children. Int J Pediatr Otorhinolaryngol.

[61] Nitschke JE, McMeeken JM, Burry HC, Matyas TA (1999). When is a change a genuine change?: a clinically meaningful interpretation of grip strength measurements in healthy and disabled women. J Hand Ther.

[62] Hageman PA, Leibowitz JM, Blanke D (1995). Age and gender effects on postural control measures. Arch Phys Med Rehabil.

[63] Bohannon RW (1997). Comfortable and maximum walking speed of adults aged 20-79 years: reference values and determinants. Age Ageing.

[64] Ihlebaek C, Eriksen HR, Ursin H (2002). Prevalence of subjective health complaints (SHC) in Norway. Scand J Public Health..

[65] Herdman M, Gudex C, Lloyd A, Janssen M, Kind P, Parkin D (2011). Development and preliminary testing of the new five-level version of EQ-5D (EQ-5D-5L). Qual Life Res.

[66] Shumway-Cook A, Horak FB (1986). Assessing the influence of sensory interaction on balance: suggestion from the field. Phys Ther.

[67] Nordin E, Moe-Nilssen R, Ramnemark A, Lundin-Olsson L (2010). Changes in step-width during dual-task walking predicts falls. Gait Posture.

[68] Bohannon RW (2015). Muscle strength: clinical and prognostic value of hand-grip dynamometry. Curr Opin Clin Nutr Metab Care.

[69] Meldrum D, Herdman S, Vance R, Murray D, Malone K, Duffy D (2015). Effectiveness of conventional versus virtual reality-based balance exercises in vestibular rehabilitation for unilateral peripheral vestibular loss: results of a randomized controlled trial. Arch Phys Med Rehabil.

[70] Holmberg J, Karlberg M, Harlacher U, Magnusson M (2005). Experience of handicap and anxiety in phobic postural vertigo. Acta Otolaryngol.

[71] Yardley L, Beech S, Zander L, Evans T, Weinman J (1998). A randomized controlled trial of exercise therapy for dizziness and vertigo in primary care. BrJGenPract..

[72] Popkirov S, Stone J, Holle-Lee D (2018). Treatment of persistent postural-perceptual dizziness (PPPD) and related disorders. Curr Treat Options Neurol.

