# Supplementary material for: Efficacy of intergrating vestibular rehabilitation and cognitive behaviour therapy in persons with persistent dizziness in primary care- a study protocol for a randomised controlled trial
Source: Trials. 2019 Oct 7;20:575. doi: 10.1186/s13063-019-3660-5 (PMC6781377; doi:10.1186/s13063-019-3660-5)
Supplement: Supplementary file 2 — Consent form for participation, in Norwegian (DOC 48 kb) [file 13063_2019_3660_MOESM2_ESM.doc]

# Forespørsel om deltakelse i forskningsprosjektet

***”Undersøkelse og behandling av pasienter med svimmelhet, i norsk primærhelsetjeneste”***

# Bakgrunn og hensikt

Dette er et spørsmål til deg om å delta i en forskningsstudie. Langvarig svimmelhet er en kjent plage, men det er fortsatt mye å lære når det gjelder hvordan helsevesenet undersøker og behandler pasienter med slike plager. Du med dine langvarige plager er derfor viktig for oss. Vi ved Høgskolen i Bergen og Bergen kommune ønsker å fremskaffe mer kunnskap om pasienter med langvarig svimmelhet, og dessuten evaluere effekten av en ny fysioterapeutisk gruppebehandling.

# Hva innebærer studien?

Dersom du blir med i studien ønsker vi at du fyller ut en del spørreskjemaer om hvordan svimmelheten plager deg, om du har andre plager relatert til svimmelheten og om den påvirker deg i hverdagen. I tillegg gjøres noen tester der vi undersøker balanse, svimmelhet, muskelstyrke -og fleksibilitet, gange, hodebevegelser og fokuseringsevne. Alle deltakere får en konsultasjon hos fysioterapeut som består av informasjon og veiledning i øvelser. Deretter vil et tilfeldig utvalg få tilbud om et gruppetiltak som består av øvelser og samtale, mens den andre gruppen trener på egen hånd. Etter 6 og 12 måneder gjennomføres tester på ny, og spørreskjema besvares.

Noen av dere vil bli forespurt om å komme til en ekstra testrunde ca. en uke etter første konsultasjon. Hensikten er å undersøke hvor nøyaktige testene som brukes er.

**Mulige fordeler og ulemper**

Det finnes pr i dag ikke et etablert behandlingstilbud til pasienter med svimmelhetsproblematikk i primærhelsetjenesten. Resultatene fra prosjektet vil derfor kunne bidra til å videreutvikle en behandlingsmodell tilpasset flere pasienters behov, og dermed gi et bedre behandlingstilbud til pasienter med svimmelhet i primærhelsetjenesten.

Øvelsene som inngår i gruppebehandlingen kan provosere svimmelheten, og dette kan oppleves ubehagelig. Slike øvelser er imidlertid nødvendige for at du skal klare å kompensere og bli bedre av svimmelheten. God informasjon i forkant av øvelsene vil gjøre at du opplever behandlingssituasjonen som trygg.

**Hva skjer med informasjonen vi har samlet om deg?**

Informasjonen som registreres om deg skal kun brukes slik som beskrevet i hensikten med studien. Alle opplysningene vil bli behandlet uten navn og fødselsnummer eller andre direkte gjenkjennende opplysninger. En kode knytter deg til dine opplysninger gjennom en navneliste. Det er kun autorisert personell knyttet til prosjektet som har adgang til navnelisten og som kan finne tilbake til deg. Ved prosjektslutt i 2018 vil alle personopplysninger om deg bli slettet. Det vil ikke være mulig å identifisere deg i resultatene av studien når disse publiseres.

**Frivillig deltakelse**Det er frivillig å delta i studien. Du kan når som helst og uten å oppgi noen grunn trekke ditt samtykke til å delta i studien. Dette vil ikke få konsekvenser for din videre behandling. Dersom du ønsker å delta, undertegner du samtykkeerklæringen på siste side. Om du nå sier ja til å delta, kan du senere trekke tilbake ditt samtykke uten at det påvirker din øvrige behandling. Dersom du senere ønsker å trekke deg eller har spørsmål til studien, kan du kontakte Lene Kristiansen (prosjektkoordinator) på mobil 94807419 eller Liv Heide Magnussen (prosjektleder) på telefon 55585631.

**Samtykke til deltakelse i studien**

Jeg er villig til å delta i studien

----------------------------------------------------------------------------------------------------------------

(Signert av prosjektdeltaker, dato)

Jeg bekrefter å ha gitt informasjon om studien

----------------------------------------------------------------------------------------------------------------

(Signert, rolle i studien, dato)
